# Extended Goal Recognition: Lessons from Magic

**DOI:** 10.3389/frai.2021.730990

**Published:** 2021-11-12

**Authors:** Peta Masters, Wally Smith, Michael Kirley

**Affiliations:** School of Computing and Information Systems, The University of Melbourne, Melbourne, VIC, Australia

**Keywords:** planning, reasoning, deception, goal recognition, SOMA, magic

## Abstract

The “science of magic” has lately emerged as a new field of study, providing valuable insights into the nature of human perception and cognition. While most of us think of magic as being all about deception and perceptual “tricks”, the craft—as documented by psychologists and professional magicians—provides a rare practical demonstration and understanding of goal recognition. For the purposes of human-aware planning, goal recognition involves predicting what a human observer is most likely to understand from a sequence of actions. Magicians perform sequences of actions with keen awareness of what an audience will understand from them and—in order to subvert it—the ability to predict precisely what an observer’s expectation is most likely to be. Magicians can do this without needing to know any personal details about their audience and without making any significant modification to their routine from one performance to the next. That is, the actions they perform are reliably interpreted by any human observer in such a way that particular (albeit erroneous) goals are predicted every time. This is achievable because people’s perception, cognition and sense-making are predictably fallible. Moreover, in the context of magic, the principles underlying human fallibility are not only well-articulated but empirically proven. In recent work we demonstrated how aspects of human cognition could be incorporated into a standard model of goal recognition, showing that—even though phenomena may be “fully observable” in that nothing prevents them from being observed—not all are noticed, not all are encoded or remembered, and few are remembered indefinitely. In the current article, we revisit those findings from a different angle. We first explore established principles from the science of magic, then recontextualise and build on our model of extended goal recognition in the context of those principles. While our extensions relate primarily to observations, this work extends and explains the definitions, showing how incidental (and apparently incidental) behaviours may significantly influence human memory and belief. We conclude by discussing additional ways in which magic can inform models of goal recognition and the light that this sheds on the persistence of conspiracy theories in the face of compelling contradictory evidence.

## 1 Introduction

Goal recognition is the problem of determining an agent’s goal by observing its behaviour. It is a long-established area of artificial intelligence (AI) research (Kautz and Allen, 1986; Charniak and Goldman, 1991; Carberry, 2001), commonly tackled for two quite different purposes: literally, to determine the observed agent’s goal; but also, once removed, to determine what an *observer*, seeing similar behaviour, is most likely to believe to be the observed agent’s goal. In recent work, Masters et al. (2021) introduced the notion of extended goal recognition (XGR) to focus on that second purpose, which is of increasing concern to practitioners in robotics and human-machine interaction, where its use falls under the heading of “interpretable behaviour” (Chakraborti and Kambhampati, 2019). In this context, we ask: when these observations are made or this sequence of actions is performed, what is a human-like observer most likely to believe?—with the important caveat that *an observer’s belief may be wrong*.

This distinction—that traditional goal recognition seeks the ground truth, while interpretable behaviour is concerned with potentially erroneous belief—is critically important but rarely acknowledged. It is easy to assume that, since both humans and goal recognition systems are trying to solve the same problem (what is the observed agent’s goal?) they ought not only to be doing it in the same way but arriving at similar conclusions. Once recognised, however, the confusion is unsurprising. Artificial intelligence, as a discipline, has always grappled with this issue. On the one hand, it sets out to achieve human-like intelligence in order to make human-like decisions; on the other, its decisions and behaviour are expected to be governed by logic and rationality. As Russell and Norvig (2013) point out (p.1), there is a big difference between thinking humanly and thinking rationally. In AI, rationality typically has the edge: it supports a strict and straightforward mathematical definition convenient both practically (e.g., for roboticists) and theoretically (i.e., for research). By comparison, human-like reasoning seems woolly and ill-defined.

Assumptions, preconceptions and false beliefs that may appear mysterious and anomalous to a computer scientist, however, are trivially consistent to those who make their livings by manipulating the beliefs of their fellow humans. Professionals in this regard include military strategists, marketers, confidence tricksters and stage magicians. In this article, we focus on the latter.

It is a magician’s job to manipulate their audience’s beliefs: to find ways of persuading it that the coin is here when really it is there, that you chose the Ace of Spades of your own free will, that the lady really has been sawn in half. Lately, moreover, the practice of magic has been analysed with renewed rigour in terms of the psychological principles at work, giving rise to an emerging field of study dubbed by Kuhn (2019) amongst others, the “science of magic”. These—and related—principles provide both the inspiration for XGR and a practical demonstration of its effectiveness. Goal recognition systems use probabilistic reasoning to predict what an observer is most likely to understand from a given sequence of actions; informally, magicians have been making similar predictions for generations, performing sequences of actions such that human observers reliably draw *erroneous* conclusions. Importantly, magicians can *rely* on the conclusions people will draw. Magic works by exploiting flaws in human reasoning that are *predictable*; and because they are predictable, they are susceptible to probabilistic/mathematical interpretation.

Behavioural scientists frame human-like reasoning of the type exploited by magicians as mistakes (Kahneman, 2011). While acknowledging that instinctive “fast” reasoning is predictable, they imply that the resultant errors can and should be corrected by applying more careful “slow” reasoning. This notion has lately been challenged, for example by King and Kay (2020). They point out that the short-cuts taken by people operating in the real world may seem like mistakes in the context of small-world problems of the type studied by economists and computer scientists, but are essential to real people operating in the real world. The question for AI—given that one frequently-cited purpose of the discipline is to try to replicate human reasoning—is this: should AI try to replicate human reasoning even when that reasoning is flawed? In the context of interpretable behaviour, XGR and indeed this paper, we suggest that it should.

In what follows, we first provide a background to the science and study of magic. We then present the underlying technical framework on which we build XGR as a series of extensions and introduce a test environment. Next, we present the lessons from magic, providing their psychological basis, their demonstrated efficacy in the context of magic, their significance to goal recognition, and formal definitions to support their technical realisation. Finally, we present our extended model and conclude by discussing further ways in which magic can inform goal recognition and the light that this sheds on issues such as the persistence of conspiracy theories and fake news even in the face of compelling contradictory evidence.

## 2 The “Science” of Magic

Magic is emerging as a rich source of insight into human cognition, as confirmed by the growing attention it has lately received from leading international scientific journals (e.g., Danek et al., 2014; Kuhn et al., 2014; Rensink and Kuhn, 2015). Work in this field offers a unique perspective to AI research in general; and to goal recognition in particular. When conducted for the purpose of human-aware planning, goal recognition involves predicting a human’s most likely belief based on observations of an agent’s behaviour. Magicians, meanwhile, practically demonstrate *precisely those factors* that determine how observable phenomena control what humans are most likely to believe.

Deception is a fruitful context for the study of goal recognition. An experiment in which someone comes to believe something that is in fact true may be able to confirm that extraneous actions caused no confusion but it is only when we see them come to believe something false that we can begin to evaluate how, why and when their (false) belief arose. Magicians are amongst a handful of professional deceivers, alongside military strategists, con-artists, crime writers and, arguably, advertising executives. Their skills extend well beyond the notion that “the speed of the hand deceives the eye”. Consider, for example, that in AI research we frequently assert that the environment must be only partially observable in order to achieve deception. Using misdirection, a magician routinely deceives even in an environment that is fully observable (in that it is *possible* for everything in the environment to be seen) by directing attention away from whatever is to be hidden and towards whatever is to be believed. Furthermore, in AI research we frequently assert that the observer must be naïve (i.e., unsuspecting) in order to achieve deception. A magician, however, reliably deceives their audience, even though that audience is fully expecting deception to occur. Unsurprisingly, practitioners have very different views about magic from those held by the general public. Amateur magicians and “muggles” in the audience tend to focus on the physical skills involved: how to palm a card successfully or conceal a coin without dropping it or giving ourselves away. But to emphasise a magician’s dexterity is like thinking the most important attribute of a concert pianist is their hand hygiene or that an actor is gifted because they are able to “learn all those lines”. Magicians, sharing information amongst themselves, tend to focus not on their physical skills but on the cognitive and decision-making aspects of their craft; and those are the topics that concern us here.

Bruno’s Anatomy of Misdirection (1978, as cited in Kuhn et al., 2014) sets out three levels of misdirection that can be seen as a progression from the lay view towards that typically held by a magician. Bruno differentiates between: *distraction*, where several things seem to be going on at the same time, creating confusion (perhaps as crude as a loud bang, one of the lay notions of misdirection); *diversion*, where although only one thing seems to be going on, such as the story the magician is telling or apparatus at which they are pointing, it is something that demands the observer’s full attention; and *relaxation*, which refers to those moments during a performance when, even without them realising it, the audience’s attention is diminished, as occurs when a trick seems to have been completed or after the same actions have been repeated multiple times (so-called “off-beats”, the more sophisticated perspective of a seasoned performer).

Lamont and Wiseman (2005) emphasise the fundamental reality that in every trick there are at least two things going on: a *method*—actions taken to make the trick happen; and an *effect*—what the audience is made to believe. Indeed, in his general theory of deception, Whaley (1982) points out that this is a fundamental reality for all strategic deception, which he maintains is always part dissimulation (hiding the real) and part simulation (showing the false). It is never enough to conceal the truth: when something is hidden, something else is necessarily shown, if only implicitly. You might hide coins in a jar, for example, showing (implicitly) that there is no money in the house.

**FIGURE 1:**
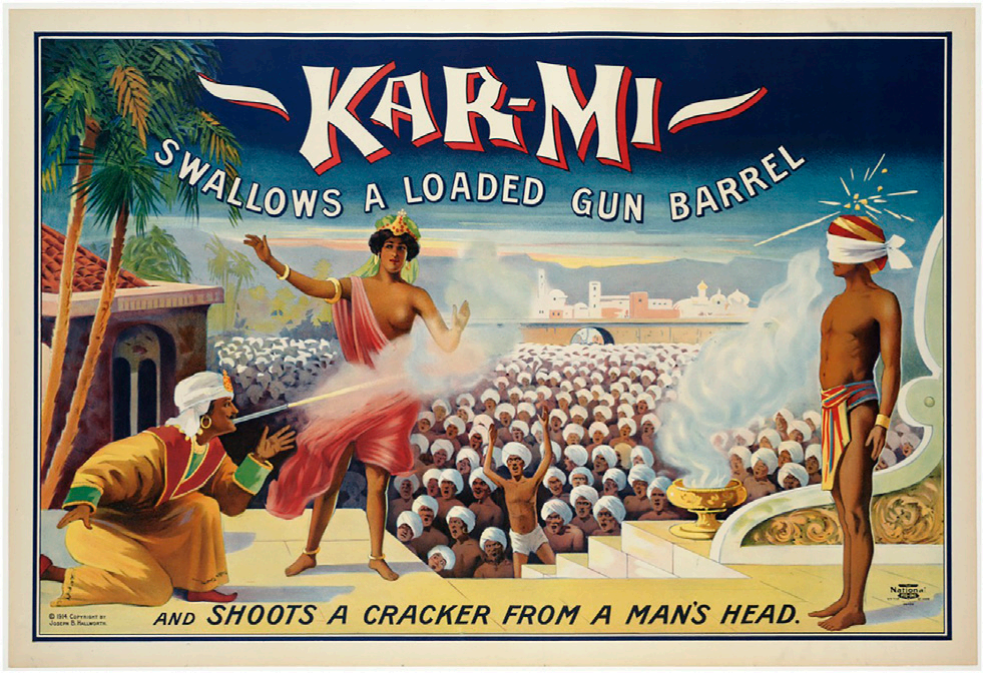
1914 poster suggesting what Kar-Mi’s audience were made to believe. Original held at Boston Library. Reproduced from the Massachusetts Collection Online under Creative Commons Licence.

With this in hand, misdirection is readily explained as anything that directs an audience’s attention away from the method and towards the effect. Lamont and Wiseman (2005) distinguish between *physical* and *psychological* misdirection. Physical misdirection, they say, can control where an observer looks either *actively* (e.g., by pointing or looking towards the effect) or *passively*, creating an area of “primary” interest around the effect through novelty, movement or contrast. More subtly, a magician can control *when* an audience looks, ensuring the method remains hidden by performing the necessary technical steps either before the effect begins or after it seems to have been completed. Psychological misdirection, on the other hand, is concerned with the observer’s *suspicion*, which can be reduced by making sure the method appears natural, that it is justified by some *ruse* (e.g., taking something from the pocket to conceal the fact that you are putting something in) or diverted by introducing false solutions or expectations. Moreover, suspicion in the method can be reduced by reinforcing the observer’s belief in the effect. One further major device considered by Lamont and Wiseman of interest to us is *reconstruction* whereby the observer is encouraged to misremember what they have seen. Only possible because people are unable to remember an extended sequence of events precisely, the magician can “remind” a spectator that the cards were shuffled, for example, and that she then freely selected a card from the deck when, in fact, the card was selected first and the deck only shuffled afterwards. Citing various well-respected magicians, the authors discuss how, by emphasis or de-emphasis, actions can be made more or less memorable—and thereby more or less likely to be available for recall when the audience later attempts to reconstruct how some particular effect could have been achieved. Figure 1 demonstrates the extent to which such practices have been found to succeed. An audience leaving Kar-mi’s performance would doubtless believe that they witnessed him swallow a gun and fire a shot out of his mouth but we can be fairly certain that this is not precisely the event that occurred.

While Bruno (1978, as cited in Kuhn et al., 2014) explains misdirection primarily in terms of controlling where the spectator looks, Lamont and Wiseman (2005) additionally link misdirection to human cognition and consider how human idiosyncrasies with respect to memory can be exploited. In their taxonomy of misdirection, Kuhn et al. (2014) take further steps towards establishing what has become known as the science of magic (SOMA), explicitly associating magical techniques with the psychological principles on which they depend. The authors categorise three types of misdirection: *perceptual* (what is observed), *memory-related* (what is remembered) and *reasoning-related* (what is believed). Perceptual misdirection may be attentional or non-attentional. That is, misdirection is not only concerned with what you notice but with what you *fail* to notice. Memory-related misdirection includes forgetting and misremembering, and reasoning-related misdirection, the most novel but least developed of the categories, incorporates a range of ruses and framing devices that may be used to deliberately engender false belief. In each case, the authors pinpoint aspects of cognition (with respect to attention, memory and reasoning) that can be targeted and manipulated and, at the lowest level, highlight the techniques magicians traditionally use to exploit them.

This mapping of specific tactics and strategies to psychological phenomena lies at the heart of SOMA and tricks have been used to shed light on significant properties of perception and attention. These include the Gestalt grouping of elements or *principle of simplicity*, whereby people assume potentially discontinuous elements are in fact continuous if they are convincingly aligned (Barnhart, 2010); *inattentional blindness*, whereby observers are unaware of events outside their attentional range; and *change blindness*, whereby observers may fail to notice even quite substantial changes if their vision is interrupted (Kuhn and Findlay, 2010). As Levin et al. (2000) point out, phenomena such as these are particularly easy to exploit because, on the whole, people are unconscious of them and assume that their visual awareness is much more acute than it really is. In addition to these “hard-wired” effects, SOMA researchers have also investigated involuntary/exogenous attention, showing how salient or newly-introduced objects command attention—creating those areas of primary focus noted (but not explained) by Lamont and Wiseman (2005).

Ortiz (2006) takes a broader view, focusing on the design and structure of a trick over the technical mechanics of its method. He emphasises that “the micro-elements of an effect (sleights, gaffs, subtleties, misdirection)” should not be regarded as an end in themselves but rather combined “on the macro level” to achieve effects that seem genuinely impossible (p.33). From this perspective, a trick is a story. It might *involve* making a coin seem to appear or disappear or move from your pocket to a cup but the experience of having witnessed something impossible depends less on individual acts than on the narrative that weaves them together, convincing the observer, for example, that the coin itself possesses magical properties. Sometimes the magician literally tells a story (e.g., by announcing that he will now saw the lady in half), sometimes implicitly (e.g., by obviously and repeatedly tearing a newspaper in half, and the halves in half again, suggesting that the paper must have ended up in shreds). Smith et al. (2016) formalise (in part) how the extended story in a magic trick works by leading observers to mentally construct a false narrative that gradually departs from reality. To do this, magicians exploit situations of incomplete information by seamlessly mixing true and false evidence, ensuring that visible and inferred elements are always consistent with the effect or “cover” story. As illusionist Derren Brown explains:

*“We are all trapped inside our own heads; and our beliefs and our understandings about the world are limited by that perspective. Which means we tell ourselves stories…So here we are in this infinite data source. There’s an infinite number of things that we could think about but we edit and delete. We choose what to think about, what to pay attention to. We make up a story—to make sense of what’s going on. And we all get it wrong”* (Brown, 2019).

## 3 Technical Framework

In this article, we show how psychological principles exploited by magicians can be formalised as extensions to existing goal recognition frameworks. Here, we introduce a generic model from (Masters and Sardina, 2019b) on which we will build to demonstrate the applicability of our approach. This model has the advantage that it can be applied online or offline, to path-planning, task-planning or motion-planning in discrete or continuous domains.

Recall that goal recognition (GR) is the problem of determining an agent’s most probable goal from observations of its behaviour. We assume a single observer (the GR system) seeking to identify a single goal, though the observed behaviours may be performed by multiple actors. We further assume that the domain supports the notion of state-to-state transitions that can be costed and that the observable phenomena within the domain, whatever they consist of (e.g., actions, states, fluents, trajectories, etc.), *are* or can be *associated with* the transitions that give rise to them.^1^

Definition 1. *A* ***generic cost-based GR problem*** *is a tuple* $P=\left\langle D,\Omega ,\vec{o},G,s,Prob\right\rangle$ *where:*

• D *is a model of the GR domain (which defines states, transitions between states and their cost);*
• Ω *is the set of all the observable phenomena in* D *;*
• $\vec{o}=o_{1},o_{2},‥,o_{n}$ *is an observation sequence,* *o*_*i*_ ∈ Ω*;*
• *G* *is the set of candidate goals;*
• *s* *is the initial state, which is fully observable; and*
• * Prob* *is the prior probability distribution across* *G* *.*

Generally, a ***plan*** *π* in D is a sequence of elements or events that imply transitions from state to state. Given a set of all such elements *E*, each element has a cost c:E↦R and the ***cost of a plan*** $cost\left(\pi \right)=\sum_{i=1}^{m}c\left(e_{i}\right)$ . A plan *π* = *e*_1_, …, *e*_*m*_ is said to ***satisfy observations*** $\vec{o}=o_{1},\ldots ,o_{n}$ , if there exists a monotonic function *f* : {1, …, *n*}↦{1, …, *m*} such that *e*_*f*(*i*)_ = *o*_*i*_ for all *i* ∈ {1, …, *n*}. That is, the ordering (in both the plan and the observation sequence) is preserved. The ***optimal (lowest) cost*** of a plan from *s* to a goal *g* ∈ *G* is denoted by *optc*(*s*, *g*) and the lowest cost plan from *s* to *g* that satisfies observations $\vec{o}$ is denoted $optc\left(s,\vec{o},g\right)$ .

The solution to P is a probability distribution which prefers those goals that best satisfy the observations. In seminal work, Ramirez and Geffner (2009) and Ramirez and Geffner (2010) introduced the notion of ***cost difference*** as a basis on which to make that distinction, being the difference between the optimal cost of a plan that satisfies observations and the optimal cost of a plan that does not. The power of the formula lies in the fact that both terms can be calculated by a classical planner while one of the key insights is that the lower a goal’s cost difference, the higher its probability (relative to other goals in the distribution).

The cost difference formula has since been analysed by others (Escudero-Martin et al., 2015; Masters and Sardina, 2017a; Masters and Sardina, 2019a) and we adopt a less computationally demanding construction than the original, proved by Masters and Sardina (2019a) to return identical results in all but one corner case:

$$costdif\left(s,\vec{o},g\right)=optc\left(s,\vec{o},g\right)-optc\left(s,g\right).$$ (1)

The ***solution*** to a problem P is given by the probability distribution at (Eq. 2) below. In words, the likelihood of a goal is inversely proportional to the cost difference. The results are then multiplied by priors (* Prob *) and normalised:

$$Pr\left(G|\vec{o}\right)=\alpha \cdot \frac{1}{e^{\beta (costdif\left(s,\vec{o},g\right))}}\cdot Prob\text{ for }g\in G,$$ (2)

where *α* is the normalisation constant and *β* is a rate parameter, which changes the shape of a distribution without changing the rankings: the lower the value of *β*, the flatter the distribution.

## 4 MindTrails

There are many standard test environments within which goal recognition systems are routinely demonstrated such as grid-navigation, logistics and blocks world (all used by Ramirez and Geffner, 2010). Although some aspects of our proposed extensions can be described in each of these contexts, we find that, individually, they are too simplistic for our needs. Before proceeding, therefore, we introduce an environment that combines elements from each of them, which we will use to provide a running example.

MindTrails (Smith et al., 2021) is a game-like environment within which:

• the overarching framework is grid-based, with navigational goals;
• shuttles deliver block-like tokens to their destinations;
• achievement of the ultimate goal depends on assembling the tokens in a particular configuration.

MindTrails was explicitly designed to afford opportunities for types of deception frequently exploited in stage magic. Critical for our purposes are:

• a fully observable environment, in that it is *possible* for all actions to be observed;
• the ability of the observed agent to execute multiple actions simultaneously;
• dependence on memory for the observer to successfully determine the observed agent’s goal;
• an opportunity for the observer to form and develop their beliefs/suspicions over time.

**FIGURE 2:**
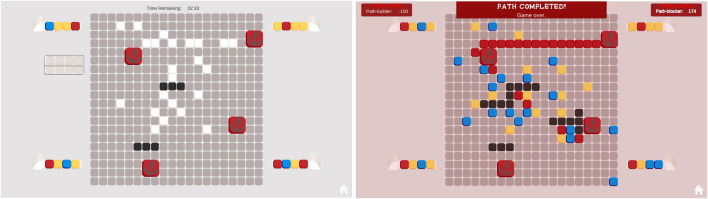
The MindTrails environment during **(left)** and on completion of a game **(right)**. There are four token generators, two shuttles (empty between turns) and a grid-like board with several anchors (in this case four). Player A uses the shuttles to ferry tokens onto the board, trying to connect two anchors with a trail of red tokens. Although the colours are visible while tokens are carried, once positioned they are all white, as if face-down. Player B tries to identify which two anchors are being connected—the black squares show where B has blocked. On completion of the game, the full state of the board is revealed.

MindTrails is a two-player game. Figure 2 shows the environment during and on completion of a typical game. There is a grid around which are positioned four *generators* producing differently-coloured *tokens*. On the grid, there are several *anchors*. Player A’s goal is to create a continuous trail of red tokens linking any two anchors. Player B’s goal is to determine which two anchors are being linked and, based on that information, to block Player A’s progress.

Player A has control of two shuttles which it uses to collect the tokens and place them face-down on the grid. The colours of the tokens are visible while they are being carried and when first positioned but, once placed, they all look the same. If an empty shuttle, however, passes over the top of a face-down token, its colour is temporarily revealed. Player B blocks by selecting particular grid locations—those it believes Player A will want to use—and rendering them unavailable.

A key feature of the game is the necessity for Player A to deceive Player B in order to win. To this end, Player A deliberately and systematically places red tokens in *false* goal zones to manipulate Player B’s beliefs.

**FIGURE 3:**
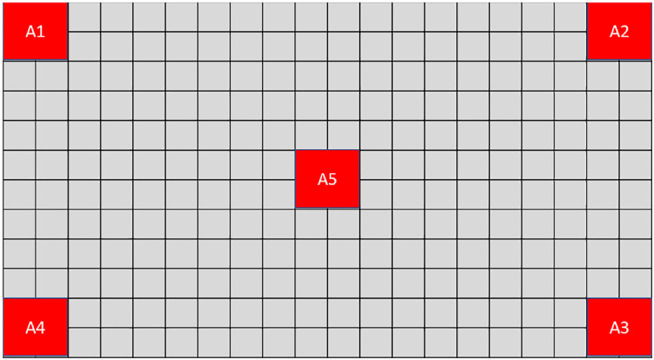
A representation of the MindTrails environment, with five anchors A1 to A5.

From a goal recognition stand-point, the set of possible goals thus comprises all possible anchor-to-anchor combinations. For current purposes, we consider the six shortest combinations only. That is, referring to the representation of the board at Figure 3, *G* = {(*A*1, *A*4), (*A*1, *A*5), (*A*2, *A*3), (*A*2, *A*5), (*A*3, *A*5), (*A*4, *A*5)}.

The observable phenomena in a MindTrails domain, Ω, are the colour/location combinations of deposited tokens. The initial state is the grid, empty except for the fixed anchors and we assume that the prior probabilities of all anchor combinations are equal. To suit the path-planning environment, we measure the cost of achieving the goal from the currently perceived state *s*_*p*_, as in (Masters and Sardina, 2019a) and as implied by the concept of path completion, which measures work left to do in the context of deceptive planning (Masters and Sardina, 2017b) where the plan to this point may or may not have been optimal.

$$costdif_{MT}\left(s,s_{p},g\right)=optc\left(s_{p},g\right)-optc\left(s,g\right)$$ (3)

As before, the lower the cost difference with respect to a path between anchors, the higher the probability that they are the goal.

## 5 Lessons From Magic

The framework in Section 3 incorporates no intentional bias. It is representative of frameworks that might be used to determine the most likely goal either of humans or machines. Though potentially used for the purpose (e.g., Masters and Sardina, 2017b), it tells us nothing—and does not claim to tell us anything—about the likely beliefs of a human agent making similar observations in a similar domain.

In this section, we examine a series of tricks and techniques that shed light on the psychological principles that underlie what people notice, remember, and believe (Kuhn et al., 2014). In each case, we suggest how the principle can be incorporated into a conventional goal recognition framework and illustrate its application by describing a worked example from MindTrails. We make two minor preliminary modifications to the Section 3 framework: first, we assume that we are always dealing with an online problem (i.e., that observations are processed incrementally and are time-sensitive); secondly, the initial state or starting point, previously *s*, is now given as *s*_*i*_ to represent the ***first remembered state*** at time-step *i* or *effective* starting point.

Importantly, our purpose is not to *defeat* the goal recognition system but to extend it in such a way that it better reflects what an average human is most likely to believe, given the observable phenomena to which they may be exposed. Inevitably, of course, this does result in a goal recognition system that can be fooled.

### 5.1 Method and Effect

For a magical experience to occur, at one or more moments in any trick, at least two things must be taking place—actions to achieve the effect that the magician wants their audience to see and actions involved in the method they want to conceal.

**Lesson 1.** *The Ambitious Card (Lorayne, 2006).^2^*

*The magician asks a spectator to select a card from the deck. He initially refuses the card as being the only unsuitable card that could have been chosen. To demonstrate why it is unsuitable, he puts the card—in this case, the four of diamonds—on top of the deck, puts another card on top of the four of diamonds, turns over the top card and it is still the four of diamonds. He puts the four in the middle of the deck, turns over the top card and it’s the four of diamonds. Whatever he does with the “ambitious card”, it always ends up on top.*

This is a trick with which the celebrated magician Dai Vernon is said to have fooled Houdini. The effect—that is, the story sold to the audience—is that the four of diamonds is an ambitious card: no matter what the magician does, it always finds its way to the top. The method—which we do not need to know precisely—tells an alternative tale in which the card is not placed where it appears to have been placed and/or not retrieved from where it appears to have been retrieved.

**FIGURE 4:**
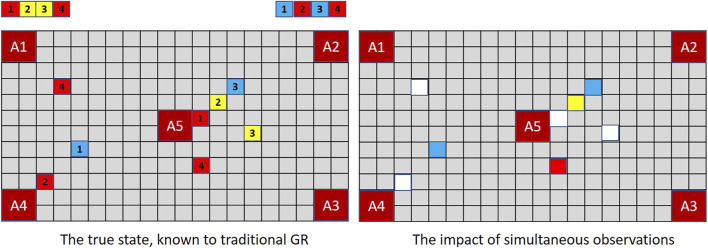
When multiple events occur simultaneously with full observability, a traditional GR system is capable of recording them all; but a human—or a GR system that supports the notion of selective attention—records only one. Thus, given shuttles delivering the combination of tokens depicted above, a traditional GR system **(left)** assesses (*A*1, *A*4) and (*A*2, *A*5) as equally probable (cost difference = −1) while an extended GR system, if selecting one of two observations randomly at each time-step, may have noticed nothing of significance so continues to rank all goals equally (cost difference = 0).

The lesson for goal recognition is depicted in Figure 4. In the real world (as in magic and MindTrails) multiple events may occur simultaneously, some that we notice and some we do not. Conventional models of goal recognition do not allow for this. They may accommodate partially observable environments such that particular actions, events or aspects of the environment are not available to the observer (e.g., Baker et al., 2011; Ramırez and Geffner, 2011) and they routinely allow for partial *sequences* of observations in that observations may be missing (i.e., not every step-on-the-path/action-in-the-plan is seen to be taken). But when an observation *is* made, everything observable at that moment and from that vantage point is assumed now known to the observer.

To be capable of reflecting the likely beliefs of a human agent, a robust goal recognition system should support the possibility that *observable events may go unnoticed*.

To achieve this, we propose replacing the sequence of individual observations $\vec{o}=o_{1},‥,o_{n}$ of Definition 1 with a sequence of sets, where each set comprises all potential observations newly available (or refreshed) at the current time-step, only one of which is ultimately encoded and remembered.

Definition 2. ***Potential observations*** *consist of a sequence of sets* $\vec{O}=O_{1},‥,O_{n}$ *, where each set* $O_{i}=\left\{o|\text{occurred at time }i\right\}$ *.*

Practically, the goal recognition system—which in an XGR context represents human-like reasoning—assembles its own observation sequence ( ${\vec{o}}_{t}$ , corresponding to the original $\vec{o}$ ) by selecting one observation at each time-step *o*_*t*_ from the set of potential observations $O_{t}$ and adding it to the sequence of observations ${\vec{o}}_{t-1}$ selected so-far.

### 5.2 Passive Misdirection

Attention is a finite resource. When multiple events occur simultaneously, people must decide—whether consciously (top-down) or unconsciously (bottom-up)—which is most deserving of consideration.

**Lesson 2.** *Penn and Teller’s Cups and Balls (Jillette and Teller, 2017).*

*Cups and balls is a well-known trick. The usual version sees the magician place three metal cups open-side down on a table then place three balls, one on top of each. The magician then seems to make the balls pass through the solid cups so that instead of being on top they are underneath, or makes the balls move invisibly from one cup to another, sometimes disappearing altogether, and so on. Magicians Penn and Teller perform an “Americanised” version of the trick using transparent plastic cups and two sizes of balls made of tin foil. There is a lot of patter and movement and then, just before the trick ends, Penn takes the three smaller balls from under the centre cup and juggles them saying, “This isn’t juggling, this is misdirection!” whereupon the centre cup is lifted to reveal a baseball.*

Penn’s blatant declaration conforms to the lay view of misdirection: some commotion takes place which literally attracts attention. The device is crude but effective because it taps into passive/exogenous attention mechanisms that are almost impossible to control (James, 1890; as cited in Ruz and Lupiáñez, 2002). This is one of the physical misdirection types mentioned by Lamont and Wiseman (2005) which creates an area of primary interest around an effect through novelty, movement or contrast.

**FIGURE 5:**
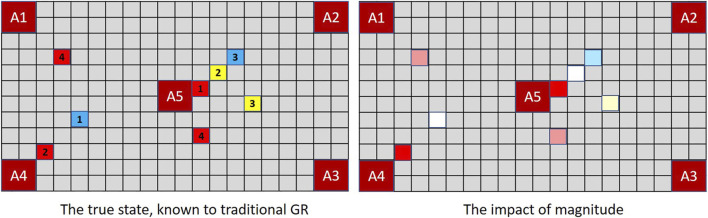
In MindTrails, red tokens are more important than non-reds. While a traditional GR system **(left)**, as before, records everything, a magnitude-aware system gives greater weight to reds, only resorting to random selection—or potentially forgetting precise locations—when magnitudes are the same. Here, after the first turn, only the reds adjacent to A4 and A5 are stored at full magnitude/clearly remembered.

Figure 5 illustrates the concept in the context of MindTrails where red tokens are more significant to an observer than non-reds. Not all observations are of equivalent salience. If an event is inherently interesting (e.g., red tokens being deposited or Penn suddenly and ostentatiously juggling), we are unlikely to notice a simultaneous event that is inherently dull (e.g., yellows being deposited or Teller momentarily putting his hand in his pocket). Yet in the observation sequence $\vec{o}=o_{1},o_{2},‥,o_{n}$ from Definition 1, there is no capacity for any one observation to carry greater weight than any other. That is, even if our proposed model did not involve selecting one out of a set of possible observations most likely to be noticed at a particular time-step $o_{t}\in O_{t}$ , it should be possible to evaluate the relative *quality* of an observation. The ability to do so—given a conventional model such as that at Section 3 or as used, say, by Sohrabi et al. (2016)—could assist in discounting a noisy observation (i.e., one that has been recorded in error), either because it is of much higher or much lower quality than those adjacent to it in the sequence.

A human-centric goal recognition system should recognise that *some observable phenomena are more noticeable than others*.

To encapsulate this notion, we say that every observable element or event in the domain has a fixed ***base magnitude*** *mag* ^∗^(*o*) for *o* ∈ Ω, which quantifies the degree to which it is inherently likely to attract attention. An explosion, for example, has a greater base magnitude than a firework, and a firework greater base magnitude than a cough. Moreover, although base magnitude is fixed, an observation *o*’s ***effective magnitude*** *mag(o)*—as perceived and later recalled by an observer—is dynamic. This means that the effective magnitude of an observation may diminish over time (see Section 5.4 on page 12). Furthermore, its initial magnitude may itself be amplified or diminished if, for example, an adversarial agent were willing to pay more for the event that gave rise to it.

To compare the relative magnitude of multiple simultaneous observations, we divide by their sum.

Definition 3. *Given a set of potential observations* $O_{t}$ *, the* ***comparative magnitude*** *of each* $o\in O_{t}$ *is given by:*

$$C\;M\left(o,O_{t}\right)=\left\{\begin{array}{cc} 0\; & \text{if}mag\left(o\right)=0\\ 0<\frac{mag\left(o\right)}{\sum_{o^{\prime }\in O_{t}}mag\left(o^{\prime }\right)}\leq 1\; & \text{otherwise}. \end{array}\right.$$ (4)

### 5.3 Active Misdirection

Selective attention in humans depends not only on bottom-up, involuntary responses to external stimuli but also on top-down processing, whereby people voluntarily focus on one thing at the expense of another. This is what we usually think of as paying attention but, although it occurs by choice, it can be manipulated; and because it can be manipulated, the extent to which it is likely to occur can be approximated.

**Lesson 3.** *The “Princess” Card Trick (Geek, 2009).*

*The magician fans out five playing cards and asks a spectator to memorise one of them. The magician closes up the fan, then goes through the cards one by one and eventually picks out one of them, looking to the spectator apparently to verify that this is definitely a match to the card they are thinking of. The magician slips that card into a trouser pocket then shows the spectator the remaining four cards and asks cheekily, “Is your card missing?” It is!*

This simple trick, which periodically appears via Facebook and chain emails—that is, the trick is so simple that even a machine can execute it!—exploits the surprising limitations of human attention and, in particular, our propensity for inattentional blindness and change blindness (e.g., Rensink and Kuhn, 2015). People may believe that they are attending perfectly well to their environment but behavioural tests reveal that anything falling outside a very narrow attentional region is neither processed nor remembered accurately. In this trick, when the magician explicitly instructs the spectator to remember one card, they fail to observe and/or remember any of the others. The principle at play is the same as that exposed in the well-known psychological experiment in which participants asked to count baseball passes fail to notice a man in a gorilla suit even though he walks directly through their line of sight (Simons and Chabris, 1999).

With limited resources, we voluntarily attend to actions and events that we deem relevant and disregard those that are not (Heuer, 1981). Contemporary models of goal recognition, however, even if performing *online* goal recognition (i.e., where observations are delivered to the system incrementally and added to a growing set or sequence) make no attempt to verify whether or not a newly added observation *makes sense*. As with magnitude (at Section 5.2 on page 9), this capability could help differentiate between plausible observations and probable noise. In our case, it is an important factor in determining which of the observable phenomena in a concurrent set $o_{i}\in O_{i}$ should be encoded.

A human-centric goal recognition system should be capable of recognising that *some observable phenomena are more relevant than others*.

To formalise this, we build on the notion of a rationality measure (RM) from (Masters and Sardina, 2019b). The documented purpose of the RM is to evaluate an agent’s future expected degree of rationality, given their past behaviour. Here, we use it to evaluate and compare the apparent rationality of the observation sequences that would result from adding each of multiple *potential* observations (each $o\in O_{t}$ ) to the *recalled* observation sequence ( ${\vec{o}}_{t-1}$ ) assembled so far. That is, given what we know, which potential observation provides the most rational continuation towards any one of the known possible goals.

Definition 4. *Given a set of possible goals* *G* *, potential observations* $O_{t}$ *, and a sequence of previously attended observations* ${\vec{o}}_{t-1}$ *, the* ***relevance*** *of an observation* $o\in O_{t}$ *is given by:*

$$rel\left(o,{\vec{o}}_{t-1},G\right)=\max_{g\in G}\frac{optc\left(s_{t-1},g\right)}{optc\left(s_{t-1},{\vec{o}}_{t-1}\cdot o,g\right)}.$$ (5)

Observe that ${\vec{o}}_{t-1}\cdot o$ in the denominator of Eq. 5 represents the observations available so far (i.e., at time-step *t* − 1) to which each newly available observation $o\in O_{t}$ is appended. Recall also that *s*_*t*−1_ is the first remembered observation—or *effective* starting point—at time-step *t*.

While the concern here is for correctness over efficiency, we note that probabilities calculated using cost difference Eq. 1 and its variations require 2|*G*| calls to the planner. Eq. 5 similarly requires 2|*G*| calls to the planner. These are not, however, *additional* calls. Rather, they piggy-back calls made to determine cost difference at the previous time-step. Furthermore, as Ramirez and Geffner (2016) point out, although planning is NP-hard, problems are nevertheless solved efficiently. This is certainly true in the context of grid-based path-planning. Moreover, in implementation, an estimated solution would often suffice.

To decide which of multiple potential observations is most likely to be attended, encoded, and available for future recall, we must put together both the top-down and bottom-up aspects of selective attention exposed in Lessons 3 and 2. That is, we propose to rely on both magnitude and relevance.

Definition 5. *Given a set of possible goals* *G* *, potential observations* $O_{t}$ *, and a sequence of previously attended observations* ${\vec{o}}_{t-1}$ *, the* ***attended observation*** *at time* *t* *,* $o_{t}\in O_{t}$ *is given by:* ^3^

$$o_{t}\left(O_{t},{\vec{o}}_{t-1},G\right)=\underset{o\in O_{t}}{\mathrm{arg max}}C\;M\left(o,O_{t}\right)\cdot rel\left(o,{\vec{o}}_{t-1},G\right).$$ (6)

**FIGURE 6:**
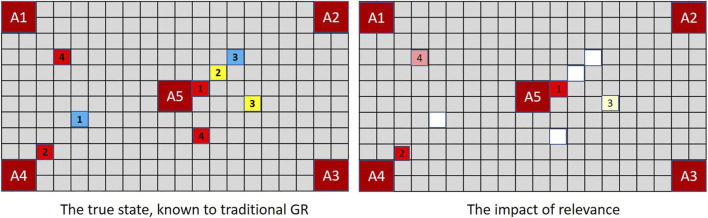
Tokens with the greatest magnitude are recorded at steps 1 and 2. At step 3, a random selection is made between non-contributory (i.e., non-red) tokens. At step 4, where two red tokens compete for attention, the most relevant red is selected: from Eq. 5, 8/7 versus 8/8.

Figure 6 shows the probable sequence of attended observations after Player A’s first turn.

### 5.4 Time Displacement

A memory-constrained agent (such as a human) cannot remember everything. Even if we initially give our full attention to a situation, we may nevertheless forget or misremember the details. Furthermore, as magicians know, this forgetfulness is almost certain to occur if our attention is overloaded and/or we are encouraged to reconstruct our memories from “alternative facts”.

**Lesson 4.** *Do As I Do (Hugard and Braue, 1949, p.83).*

*This trick is performed using two packs of cards. The magician instructs a spectator to copy everything they do. The magician shuffles one pack, and the spectator shuffles the other in the same way, then they swap packs and both shuffle again. The magician secretly chooses a card and replaces it in their pack, and the spectator does likewise. They continue to cut, swap, shuffle and reorganise the cards, both following the same procedure exactly. Finally, they both retrieve the cards they had each freely chosen at the outset to find that, completely voluntarily, they had both chosen exactly the same card!*

Critical to concealing the method for this trick is the fact that the spectator fails to remember, or later thinks irrelevant, which pack they chose their card from in the first place. This memory failure is virtually guaranteed: first, by the lengthy process of shuffling, cutting and so on, which continues long after the card has been selected, and second, owing to the level of attention required (as already noted, a severely limited resource) to precisely imitate what the magician is doing.

As Kuhn et al. (2014) remark, if events are sufficiently complex, critical events are forgotten against the noise of others; a point also made by the conjuring theorist Fitzkee (1945). In this case, moreover, the “magic” has taken place at the start of the trick, temporally distant from the reveal and, as Ortiz (2006) observes, “If you can mislead them as to when [an] effect happened, you’ll guarantee they’ll never figure out how it happened” (p.195).

**FIGURE 7:**
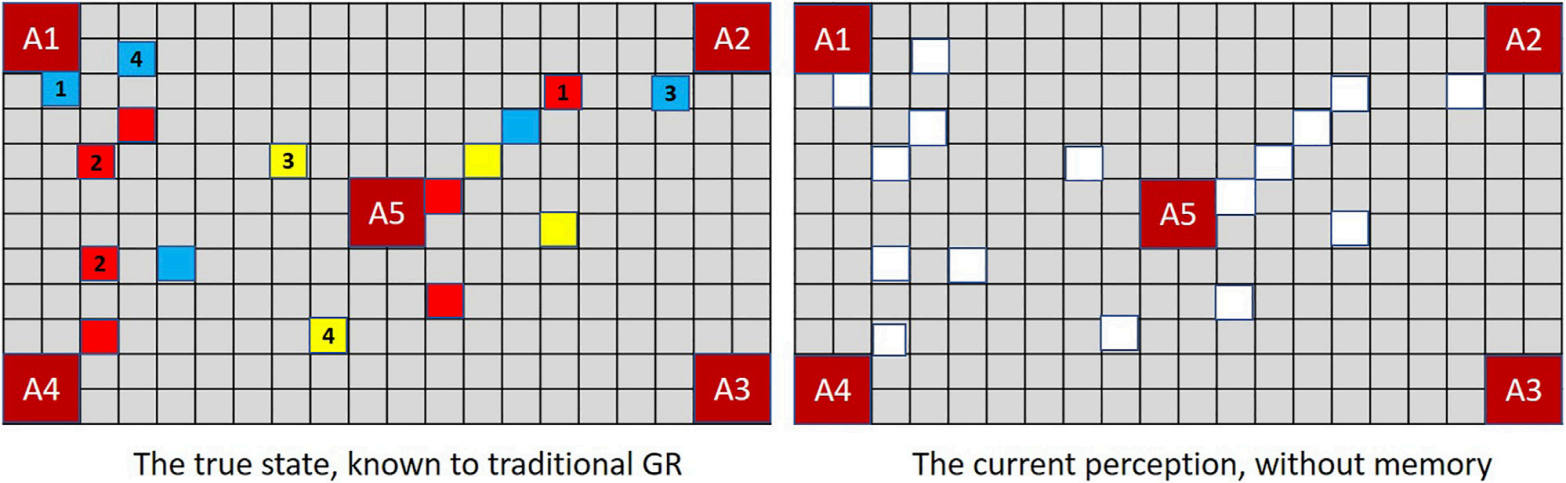
A traditional GR system **(left)** records the MindTrails board correctly, as if all tokens were face-up. A human—and extended GR—depends on *currently* perceived data (all face-down) and recalled observations, which are subject to decay. Only those perceived most recently and with the greatest magnitude are likely to persist.

The more observations a person makes, the more likely they are to forget the ones they made previously. Yet in the context of goal recognition we typically assume that, once registered, all observations remain available indefinitely. This mismatch between machine and human-like processing can critically derail the goal recognition process in an XGR context. Figure 7 contrasts the full recall of a typical GR system with the actual vision that confronts a human or extended GR during a MindTrails game.

This effect is particularly significant when we consider the special status typically given to the initial state. Recall from Section 3 that the core principle of cost-based goal recognition is that of cost difference (Eq. 1): the difference between the cost of an optimal plan versus the lowest cost achievable given actions that have been observed actually to have occurred. But to estimate either of those parameters, we need to know *which state the agent started from*. Now, contemporary models of goal recognition such as that at Section 3 typically make the strong assumption that the initial state is fully observable (e.g., Ramirez and Geffner, 2010; Vered et al., 2016; Pereira et al., 2020). But what if the human observer has forgotten the initial state and is instead referencing their first *remembered* state? Now the goal recognition system and the human may be trying to solve completely different problems.

To perform human-like reasoning, *the quality of stored observations should not be fixed; it should decay over time*.

Two factors already mentioned facilitate the above. First, recall from the beginning of the current section that, in our extended framework, the initial state, previously *s*, is given as *s*_*i*_ to represent the first *remembered* state at time-step *i*. Secondly, at Section 5.2 on page 9, we introduced the notion of magnitude as a property of each observable entity in the domain, to represent an observation’s memorability. With these in hand, our model adopts a conventional implementation of decay (familiar from work such as (Van Dyke Parunak et al., 2007) involving pheromones).

Given an observation sequence ${\vec{o}}_{t}=o_{1},o_{2},‥o_{n}$ , at every *subsequent* time-step, *t* + 1, *t* + 2, *etc*., the effective magnitude of each element *mag*(*o*_*i*_) is multiplied by a decay factor $\delta <1\in R^{+}$ . If magnitude drops below some threshold of negligibility *ϵ*, the observation is removed from the sequence at the next time-step.

Definition 6. *Given a decay factor* *δ* *, observations so-far attended and remembered* ${\vec{o}}_{t-1}$ *and the most recently attended observation* *o*_*t*_ *, the currently* ***remembered observation sequence*** *at time* *t* *is given by:*

$${\vec{o}}_{t}={\vec{o}}_{t-1}\cdot o_{t}|\left(mag\left(o\right)\cdot \delta \right)>\epsilon \text{ for each }o\in {\vec{o}}_{t-1}.$$ (7)

Thus, ${\vec{o}}_{t}$ may represent a different observation sequence at every time-step. Typically, the sequence is first-in-first-out. That is, oldest observations are forgotten first. If an observation is particularly intense, however (i.e., has excessive initial magnitude), it may persist long after more standard observations have been forgotten so that an observation sequence at one time-step may even have different cardinality from that at another.

### 5.5 The Ruse

We have so far considered the intrinsic memorability of observable actions and events (Section 5.2), the likelihood of them being attended (Section 5.3) and their propensity to decay (Section 5.4). We noted that the subject of selective attention is partly determined by its relevance. The ruse represents a corollary to that. It is one of the mechanisms whereby a magician can make an observation seem irrelevant and thereby renders it forgettable.

**Lesson 5.** *The Bullet Catch (Moretti, 1980).*

*The magician introduces an assistant with a gun and a box of bullets and claims that when the gun is fired at him he will be able to catch the bullet in his mouth. To demonstrate that no trickery is involved, he opens the box of bullets, removing its cardboard sleeve, and invites a spectator to choose three bullets. He then holds out a container with three pens and asks the spectator to choose one and make a distinguishing mark on each of the bullets. The bullets are dropped into a clear wine glass. He invites the spectator to choose one bullet, which the assistant fires into a metal can to demonstrate that the gun is real. The spectator now chooses another bullet to be fired at the magician. The assistant shoots! The magician is uninjured but immediately appears to have caught something in his mouth which he spits out onto a small silver plate. As the spectator verifies, it is the very bullet that was fired from the gun!*

“The ruse is a plausible, but untrue, reason, or action conveying a reason, for concealing the true purpose for doing something” which “makes it possible for the magician to do an unnatural thing naturally” (Fitzkee, 1945). The actions involved in a ruse may receive attention at the time they take place (everyone saw the magician handle the cardboard sleeve around the bullets, the container of pens, the wine glass, the plate) but what he did with each prop seemed to have a valid purpose at the time and is likely to be forgotten once that purpose is complete. What is surprising here is that spectators, who are in a hyper-vigilant mode when watching a magic performance, are so willing to disregard and forget incidental events. This is to do with the way memories are stored and retrieved. Whereas once it was thought our memories were laid down almost like video recordings (Chabris and Simons, 2009), available to “replay” under hypnosis, for example; we now believe that each time we recall a memory we reconstruct it from mental representations that are highly abstracted and edited down according to perceived relevance (Loftus and Palmer, 1996).

Two lessons are available to us from the ruse. First, once a sub-goal (e.g., “mark the chosen bullets”) has been realised, the actions required to achieve it are quickly forgotten so may be pruned from the observation sequence. To fully incorporate this notion into our framework, however: first, it must be possible to decompose the goals into sub-goals; and second, the framework itself must be capable of supporting the concept which, in our case, would require non-trivial modification (see discussion, p.19).

There is, however, a second more general lesson to be taken from this trick that we will focus on. Researchers have recognised that a robust goal recognition system ought to be capable of dealing with noisy observations (Sohrabi et al., 2016) and with apparently redundant/excessively suboptimal behaviour (Masters and Sardina, 2021). Audience responses to the ruse suggest that humans prune such observations out entirely, treating apparently redundant behaviour as irrelevant; and irrelevant behaviour as forgettable.

For the purposes of human-like modelling, *the less relevant an observation seems to be, the more quickly it is forgotten*.

We have already defined relevance (Definition 4) in order to model selective attention. We re-use that definition here to formalise an observation’s forgettability. To achieve this, rather than one uniform rate of decay *δ*, we calculate a rate of decay for each observation, relative to its perceived relevance.

Definition 7. *Given a default decay factor* *δ* *, goals* *G* *, potential observations* $O_{t}$ *, and a sequence of previously attended observations* ${\vec{o}}_{t-1}$ *, the* ***decay factor*** *of each observation* $o\in O_{t}$ *is given by* $rel\left(o,{\vec{o}}_{t-1},G\right)\cdot \delta$ *.*

A fully relevant observation (i.e., one that, when concatenated to observations already made, conforms to an optimal plan for one of the goals), decays at the default rate. The less relevant an observation, the lower the decay factor and therefore—through multiplication—the faster its corresponding rate of decay. Practically, to incorporate the notion of forgettability, *all* observations must be tested for relevance, not only those that may initially be overlooked as a result of selective attention (as at Section 5.3).

**FIGURE 8:**
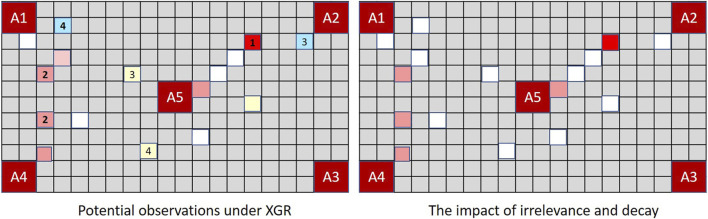
The board on the left shows observations *potentially* recalled under XGR following the second turn (true state as depicted in Figure 7). When dimly remembered observations *and* those perceived to be irrelevant determine the rate of decay, the stored state of a human or extended GR system resembles the figure on the right. Adopting a worst case assumption such that misremembered and unobserved tokens are incorporated into Player B’s assessment as potential reds, a GR system extended to *forget* ranks (*A*2, *A*5) as more likely than other possible goals (cost difference = − 5). A traditional GR system, which—since every action has been fully observable—has been able to track the complete state, now ranks (A1, A4) as most probable with a cost difference of − 3, as against the *actual* cost difference for (*A*2, *A*5) of 7 − 8 = − 1.

Figure 8 demonstrates the implications in the context of MindTrails.

### 5.6 Priming

People see what they expect to see. One approach to magic then—in line with the observation that we become trapped in the stories we tell ourselves (Brown, 2019)—is to start them off in the wrong direction: prime them about what to expect, maintain their false belief, then surprise them when the reality turns out to be something completely different.

**Lesson 6.** *The Vanishing Ball (Kuhn, 2006).*

*The magician holds a ball in his hand. He tosses it in the air and catches it, then tosses it in the air and catches it, then tosses it in the air and…but wait—it’s gone!*

In this trick, repetition primes the audience to expect that the third ball toss will proceed exactly like the second. The ball will go up in the air and fall back down again. The repetition also numbs us somewhat: we know what is going to happen so we pay less attention. Most people, seeing this trick, report seeing the ball leave the magician’s hand on the last throw but never come down (Kuhn and Land, 2006). This “miracle” is achieved by exploiting an aspect of *confirmation bias* (Wason, 1968): our tendency to notice, seek out and remember information which supports our existing beliefs but to avoid and forget information that might contradict them. Confirmation bias depends on two conditions, both present in Lesson 6: first, evidence is partly hidden or too broad in scope to be fully apprehended; and second, we feel justified to hold beliefs about partially observed things.

Here, the ball is first, partly hidden when in the magician’s hand but second, we have the reasonable expectation that it will not dematerialise just because we cannot see it.

Probabilistic goal recognition systems already accommodate confirmation bias to a degree. Observation sequences are typically incomplete and expectation is factored into Bayes’ Rule (on which probabilistic solutions are commonly based) as *prior probability*. Generically:

$$\mathrm{Pr}\left(A|B\right)=\frac{\mathrm{Pr}\left(B|A\right)\times Pr\left(A\right)}{Pr\left(B\right)}.$$

That is, the probability of a goal (A) given the evidence (B) is the probability of the evidence given the goal multiplied by the *prior probability of the goal* and divided by the probability of the evidence. The framework at Section 3, on which we are building, references the prior probability distribution across goals (* Prob *) appropriately in Eq. 2. However, despite the demonstrated importance of incorporating and updating priors in systems intended to model human-like reasoning (Baker et al., 2009; Baker et al., 2011), often those updates seem not to occur (e.g., Ramirez and Geffner, 2010; Masters and Sardina, 2019a). Instead, priors are initially assumed equal, then remain frozen: which means they cancel out and can be ignored. And this is the case even in online scenarios which implicitly consider each new observation with reference to those that have gone before. The conundrum for goal recognition lies in the fact that priors handled *more* carefully are *more* consistent with human-like reasoning but may generate *less* accurate results.^4^ As Brown (2019) warns, “We make up a story to make sense of what’s going on. And we all get it wrong”.^5^

To reflect the current beliefs of a human, prior probabilities—which represent *previously*-held beliefs—*must be kept up-to-date*, even if the system thereby seems to return the “wrong” answer.

To incorporate expectation into the model, we must ensure that prior probabilities are meaningfully considered. To achieve this, we will replace the * Prob * parameter of Eq. 2—which represents the probability distribution across goals supplied as part of the problem definition—with an explicit reference to the probability distribution calculated at the immediately preceding time-step.

$$Pr\left(G|\vec{O}\right)=\alpha \cdot \frac{1}{e^{\beta \left(optc\left({\vec{o}}_{t},g\right)-optc\left(s_{t},g\right)\right)}}\cdot Pr\left(g|{\vec{o}}_{t-1}\right)\text{ for }g\in G,$$ (8)

where *α* is a normalisation constant, *β* is the confidence parameter (discussed below) and $\vec{O}$ , recall, is the sequence of *all* sets of observations, from which time-sensitive sequences ${\vec{o}}_{t}$ and ${\vec{o}}_{t-1}$ can be extrapolated.

**FIGURE 9:**
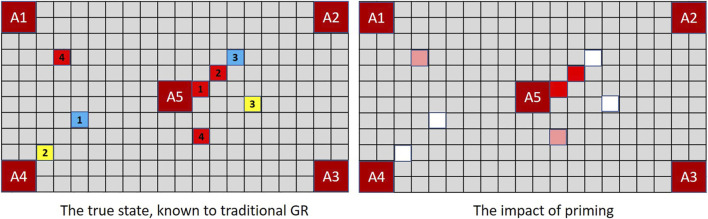
A deceptive Player A, programmed to exploit a human or extended GR system’s confirmation bias, plays its first two red tokens on an optimal path linking (*A*2, *A*5). Combined with the impacts of misremembered and forgotten tokens, Player B now assesses the probability of (*A*2, *A*5) significantly higher than that of (*A*1, *A*4), with cost differences of − 3 and 0 respectively. When probabilities are reassessed after Player A’s next turn, the differential will be hard to shake.

To demonstrate how prior probabilities can be exploited by a deceptive agent, Figure 9 shows a slight reworking of Player A’s first move in MindTrails, given the same shuttle contents as those used at Figure 4.

### 5.7 Convincers

Confirmation bias inclines human observers to seek out confirmatory evidence for their previously-held beliefs. “Convincers” are one method in a magician’s arsenal for providing such evidence. Essentially, when our expectations are confirmed, confidence increases, our next prediction is made with even more certainty; and the effect can snowball.

**Lesson 7.** *Dai Vernon’s Triumph (Ammar, 1990).*

*A spectator is invited to pick a card then return it to the deck. The magician cuts the cards, turns one half face-up, leaves the others face-down and “riffle” shuffles the two halves together in plain sight so that the cards must be completely mixed up, some face-up, others face-down. The magician then cuts the cards a few times allowing us to notice that, as expected, sometimes the card he cuts to is face-up, other times face-down. Incredibly, however, after a few more simple cuts and having passed his hand across the deck, thereby casting a mysterious shadow…the pack is spread out to reveal that all the cards are now face-down except one: the spectator’s chosen card!*

In this trick, the spectator is primed by the shuffle to believe that the deck of cards is completely mixed up, even though its actual condition is hidden to them. Whatever the method, the *magic* depends on confirmation bias whereby the audience continues to believe what they were primed to believe at the start. To reinforce the effect, the magician lets the spectator see face-up and face-down cards distributed apparently at random through the “mixed” deck. These are the *convincers*: brief and highly selective exposures to hidden aspects of the situation that are consistent with what the spectator erroneously believes (Ortiz, 1994). Most effective are *accidental* convincers, as used here, where the exposure seems unplanned and as if not even realised by the magician, making them seem to provide independent support for the spectator’s belief. This incidental quality, combined with our strong bias to readily accept confirmatory information, prevents suspicion arising about a convincer’s brevity or possible selectiveness. A similar technique has been noted in other contexts: “With respect to deception, one overwhelming conclusion stands out: It is far easier to lead a target astray by reinforcing the target’s existing beliefs…than to persuade a target to change his or her mind” (Heuer, 1981, p.298).

**FIGURE 10:**
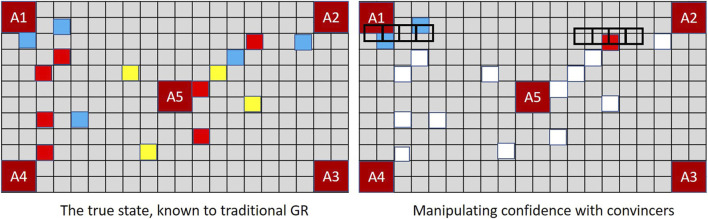
In MindTrails, when an empty shuttle passes over a token its colour is momentarily revealed, reminding Player B of events that might otherwise be forgotten. Depending on their state of decay, these may be restored to their full magnitude or perceived as entirely new observations. A new positive observation at the previously suspected goal increases confidence.

MindTrails directly supports the notion of convincers by revealing the colour of face-down tokens only when an empty shuttle passes over the top of them. Having deposited their tokens, empty shuttles must move to the side of the board making this momentary reveal seem accidental. As shown in Figure 10, if Player A deliberately passes its shuttles over red tokens on at a false goal and/or non-reds at a true goal, Player B updates its observations—and hence its probabilities—accordingly.

Probabilistic goal recognition systems have the capacity to vary the confidence with which predictions are made, using a rate parameter to change the shape of a distribution without changing goal rankings. Ramirez and Geffner (2010) leave adjustment of the parameter to an assumed operator. In (Masters and Sardina, 2021) the parameter self-adjusts but always negatively, when the actions of the observed agent seem non-rational.

In a goal recognition system designed to mirror human belief, *confidence should increase when predictions are confirmed*.

To formalise the concept, we propose to measure confidence in terms of progress made towards the goal previously thought to be most probable (i.e., the goal estimated to have the highest prior probability at the previous time-step). Remembering that *o*_*i*_ is the most recently added observation at time-step *i*, the following definition measures the difference between optimal expected and actual progress.

Definition 8. *Given a goal* $\hat{g}\in G$ *such that* $Pr\left(\hat{g}|{\vec{o}}_{t-1}\right)\geq Pr\left(g|{\vec{o}}_{t-1}\right)$ *for all* $g\in G\backslash \left\{\hat{g}\right\}$ *(i.e.,* $\hat{g}$ *was the most probable goal at time-step* *t* − 1*),* ***confidence*** *is given by:*

$$conf\left(o_{t},o_{t-1},\hat{g}\right)=e^{optc\left(o_{t-1},\hat{g}\right)-optc\left(o_{t},\hat{g}\right)-optc\left(o_{t-1},o_{t}\right)}.$$ (9)

Under Definition 8, when the most recent observation *o*_*t*_ is part of an optimal plan for the expected goal $\hat{g}$ (based on the previous observation *o*_*t*−1_), confidence is maximised; if the plan has diverged from expectation, confidence is correspondingly reduced.

Recall that, in the previous section, Eq. 8 incorporates prior probabilities and also references a confidence parameter *β*. To more completely capture those aspects of confirmation bias exposed in Lessons 6 and 7, we propose to make that parameter self-modulating, explicitly set to the observer’s current level of confidence as per Definition 8. That is, with reference to Eq. 8, $\beta =conf\left(o_{t},o_{t-1},\hat{g}\right)$ .

### 5.8 Last Lessons

We conclude this section by mentioning two lessons that have already been learnt: one already applied routinely in the context of goal recognition, the other already applied in a motion-planning domain.

**Lesson 8.** *Sawing the Lady in Half. The lady climbs into the box. Her head is at one end, her feet are at the other end. We assume her body is in the middle. And then the magician takes out his saw…*

This trick depends on the principle of simplicity whereby we assume continuity because the two ends of the lady’s body seem to line up. In a goal recognition context, we apply this principle routinely to make sense of observation sequences within which some, often many, observations are missing. Typically, we assume observations can be “stitched together” by whichever sequence of actions would be most economical.

**Lesson 9.** *The Disappearing Coin. The magician takes a coin in his right hand, then passes it to his left hand. In case we might suspect that he did not really pass the coin, he shows us that his right hand is empty. But then he reveals that his left hand is empty too!*

In this trick, the magician pre-emptively removes one possible explanation for the effect before revealing that the object has vanished. The explanation is discounted even before it has been formed. The principle at work here has been incorporated into goal recognition by Vered and Kaminka (2017) who demonstrate it in the context of motion-planning. If the observed agent changes course so as to move away from a possible goal by a sufficiently extreme angle, that goal is pruned from the set of possible goals: the agent has demonstrated that they are *not* aiming for that goal so it is completely removed from consideration.

## 6 Extended Goal Recognition

We now bring together what we have learnt under one framework, a modified version of that presented in Section 3, which incorporates the extensions that we have been discussing.

XGR is the problem of determining from observed behaviour, not necessarily the most likely goal (though it may be), but the goal that will be *believed* most likely by a predictably fallible, human-like observer. Unlike traditional GR, XGR supports several additional notions: 1) observation sequences are time-sensitive; 2) an agent may be faced with multiple competing observable phenomena, *only one of which* it is capable of fully attending at any one time; 3) previously remembered observations may be forgotten. Thus, the agent’s sphere of operation is partially observable, not because information is withheld or because the agent’s sensors are faulty, but because the model aims to capture a boundedly-rational agent unable to process and retain all the observations made available. That is, while the domain itself may be fully observable, it is not necessarily fully observed.

Definition 9. *An* ***extended GR problem*** *is a tuple* $P_{\;x}=\left\langle D,\Omega ,{mag}^{*},\delta ,\vec{O},G,s_{0},Prob\right\rangle$ *where:*

• D *is a model of the GR domain which defines states, transitions between states and their cost;*
• Ω *is the set of all observable phenomena in the domain;*
• ${mag}^{*}:\Omega \mapsto R$ *is the base magnitude of each observable;*
• $\delta <1\in R^{+}$ *is the default decay factor.*
• $\vec{O}=O_{1},O_{2},‥O_{n}$ *is a time-ordered sequence of sets, where each set* $O_{i}\subseteq \Omega$ *comprises all observable phenomena available to the agent’s sensors at a particular time-step* *i* ∈ {1, 2, …, *n*}*;*
• *G* is the set of candidate goals;
• *s*_0_ is the initial observation, which includes all cost-relevant data; and
• * Prob* *is the prior probability distribution over* *G* *.*

Observe that, as for Definition 1, we assume that the domain supports costed state-to-state transitions that can be associated with observable phenomena. We now also assume that observations include sufficient information for ongoing costs to be calculated. For example, if costs within the domain are calculated in terms of distance, then each observation is assumed to include positional data; if costs depend on spending, observations include remaining funds. This proviso enables us to calculate optimal costs that would previously have been measured by reference to a fully observable initial state by reference instead to the first remembered observation.

The solution to an XGR problem is a probability distribution across goals or—more properly, since sets of observations are delivered incrementally—a *sequence* of probability distributions calculated using Eq. 8, for convenience reproduced here.

$$Pr\left(G|\vec{O}\right)=\alpha \cdot \frac{1}{e^{\beta \left(optc\left({\vec{o}}_{t},g\right)-optc\left(s_{t},g\right)\right)}}\cdot Pr\left(g|{\vec{o}}_{t-1}\right)\text{ for }g\in G,$$ (8)

which now explicitly incorporates prior probabilities by reference to the probability distribution calculated at the previous time-step, $Pr\left(g|{\vec{o}}_{t-1}\right)$ and in which, moreover, the *β* parameter has been made self-modulating to represent the observer’s confidence.

Definition 9 differs from Definition 1 in its representation of the following features.

1) It explicitly describes an *online* problem. That is, observations are delivered incrementally at distinct time-steps.
2) Each observable element *o* ∈ Ω has a base magnitude mag*(*o*), which may be infinite but is otherwise subject to decay at a rate determined by an individualised decay factor calculated from the default. Thus, at any given time-step, an observation has an *effective* magnitude *mag*(*o*), which may differ from its base magnitude.
3) Instead of a sequence of individual observations $\vec{o}=o_{1},‥,o_{n}$ , $\vec{O}=O_{1},‥,O_{n}$ is a sequence of *sets*, where each set $O_{i}=\left\{o|\text{occurred at time }i\right\}$ . That is, each set comprises all potential observations newly available (or refreshed) at the current time-step, only one of which is ultimately encoded and remembered.
4) There is no initial state as such. *s*_0_ is the first *remembered* state. Subsequently, *s*_*t*_ is taken to represent the first remembered state at time-step *t*.

Given these modifications, which could be implemented as extensions to many GR frameworks—not only that of Section 3—all and any of the enhancements proposed in Section 5 may be implemented.

To conclude this section, we return to our running MindTrails example. Although we have not, as yet, undertaken formal experimental evaluation of the model, we are nevertheless able to calculate indicative results. Table 1 compares probabilities calculated under the traditional GR model of Section 3 with those now available to us under the extended model. We calculated probabilities on completion of each turn, assuming fully observed states as shown at Figure 4 (p.9) and Figure 10 (p.17). As the table shows, impeded by the limitations with respect to attention and memory outlined in this paper, extended GR predicts a trail at (A2, A5), its conviction reinforced by taking probabilities at Turn 1 as priors for Turn 2. Traditional GR meanwhile, impervious to manipulation, more narrowly (but correctly) predicts a trail at (A1, A4).

**TABLE 1:** A representative calculation to illustrate the differences between traditional and extended GR. (Figures in bold indicate the goals with the highest probability. Under traditional GR, there is no clear winner after Turn 1).

	Traditional GR		Extended GR	
**Goal**	**Turn 1**	**Turn 2**	**Turn 1**	**Turn 2**
(A1,A4)	0.17	**0.21**	0.15	0.16
(A1,A5)	0.17	0.16	0.15	0.14
(A2,A3)	0.15	0.14	0.13	0.10
(A2,A5)	0.17	0.16	**0.22**	**0.30**
(A3,A5)	0.15	0.16	0.15	0.11
(A4,A5)	0.17	0.14	0.17	0.16

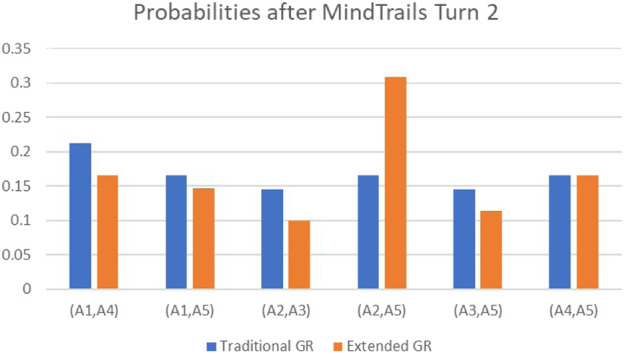
 Table 1 shows probabilities calculated for each MindTrails goal (i.e., anchor pairs that may be linked) taking the game state as at Figure 4 (p.9) for Turn 1 and as at Figure 10 (p.17) for Turn 2. For traditional GR, probabilities were calculated using Eq. 2, assuming access to all available data (i.e., actual token colours and locations). For extended GR, we used Eq. 8, assuming observations limited in line with principles established in Lessons 1 to 7 and under the worst-case assumption (i.e., unknown tokens may be red). Under these conditions, extended GR identifies (A2, A5) as the most probable goal after Turn 1 and, with Turn 1 probabilities used as prior probabilities for calculations after Turn 2 (for extended GR but remaining frozen and equal under the traditional model), the result is accentuated, as seen in the accompanying chart.

## 7 Conclusion

In this paper we have considered how magic, with its deep understanding of the idiosyncrasies surrounding human cognition and belief-formation, can inform the theory and practice of goal recognition. Our contribution is not a framework that performs goal recognition “better” than other models. If anything, our framework is less likely to determine the observed agent’s most likely goal than alternative contemporary models are able to do. Rather, we have aimed to present a framework that more accurately simulates human-like reasoning, even where that reasoning may lead to error.

By analysis of nine tricks, we have shown that, with relatively few modifications, a traditional model of goal recognition can be transformed into one capable of interpreting an observed agent’s behaviour in a human-like fashion. We have necessarily demonstrated the extensions in relation to a particular framework but, in presenting them as we have, aimed to show that they could equally be applied, piecemeal or in their entirety, to many other contemporary models. The lessons from magic relate to attention, memory and reasoning. Correspondingly, the majority of the extensions concern the treatment of observations—which observable phenomena are most likely to be seen? and how long are they likely to be remembered?—notions almost universally applicable, whichever model of goal recognition is under consideration.

The lessons have consequences and their application in the context of goal recognition go a long way towards explaining the persistence of conspiracy theories and fake news, even in the face of contradictory evidence. As we have seen, priming impacts expectation, which we model as prior probability; convincers impact confidence; and the two factors work in tandem. An exaggeratedly confident prediction accentuates the probability of the most probable goal. If, owing to the confidence value (*β* in Eq. 8), an observer is sufficiently certain of a particular goal, the probability of that goal approaches *1*. At the next time-step, that confident assessment becomes a *prior* probability. Now the probability of other goals is pushed close to zero with the result that—regardless of implications arising from the most recent observation—they may be overlooked. This matches our understanding from behavioural science and other commentators (Heuer, 1981) and is precisely the effect noted in (Masters et al., 2021), where in a fully observable path-planning domain, we find that XGR predicts as most likely a false goal, even after the real but “disbelieved” goal has been achieved.

We do not claim to have delivered a complete or definitive account. Magic has a long, rich history and, no doubt, we have barely scratched the surface. Neither are we the first to consider principles from magic in the context of AI. The relationship been magic tricks and mathematical concepts has long been noted and explored (Gardner, 1956; Diaconis and Graham, 2011). Specific connections to computer algorithms have been made, especially for teaching purposes (Curzon and McOwan, 2008). Most relevant, direct parallels have been drawn to AI with machines designing tricks (Williams and McOwan, 2016) and in the formalisation of surprise using Bayesian predictive coding (Grassi and Bartels, 2021). To our knowledge, however, we are the first to apply them in a principled way to the problem of goal recognition.

Looking ahead, we have noted the interplay between goals—or *believed* goals—and the way that people reason about observations (p.14). Owing to our propensity to dismiss and/or quickly forget actions that seem to have been accounted for, magicians make considerable use of sub-goals. While early goal recognition models based on an event hierarchy natively incorporate sub-goals (e.g., Kautz and Allen, 1986) as do those based on probabilistic grammars (e.g., Geib and Goldman, 2009; Geib et al., 2016), they are not readily combined with the cost-based reasoning used in this paper. Meanwhile *landmarks* (i.e., actions or events that must occur in order for a particular goal to be achieved), though ostensibly similar to sub-goals (Masters and Vered, 2021), are not associated with sub-sequences of behaviour and it is these sub-sequences that we would typically wish to prune. We see the accommodation of sub-goals into our framework as an interesting challenge for future work, perhaps building on (Pattison and Long, 2010).

Findings from behavioural economists have found their way into AI research, in particular with revelations about our inability to properly execute probabilistic reasoning (Kahneman, 2011). This is eminently respectable work with a strong mathematical foundation and has been eagerly taken up by computer scientists (Rossi and Loreggia, 2019; Bonnefon and Rahwan, 2020, etc.). Magic has a different pedigree; to the uninitiated, seemingly haphazard and undisciplined. We submit, however, that revelations from stage magicians, in relation to human cognition and people’s lack of insight with respect to their cognitive limitations, are equally compelling, certainly more disturbing and—when imported into goal recognition systems for the purpose of investigating and supporting human-robot interaction and applications involving interpretable behaviour—potentially more useful. The secrets shared between magicians are just beginning to be mathematically formalised while the principles that underlie them continue to be empirically proven night after night in performances around the world.

## Data Availability

The original contributions presented in the study are included in the article. Further inquiries can be directed to the corresponding author.

## Footnotes

1 Technical detail supporting the current article, including a preliminary version of the XGR model, appeared previously in Masters et al. (2021).

2 For the benefit of those wishing to see the tricks performed, note that most of the lesson citations link to YouTube videos.

3 Since the definition is comparative (we seek the maximum value), use of multiplication is somewhat arbitrary. It does, however, conveniently constrain the result within bounds [0,1]. In implementation, multiple observations might return the same maximal result in which case selection could be randomised.

4 For analysis of this issue, refer to Masters et al. (2021).

5 Quoted more fully on p.5.
